# The impact of COVID-19 on the eating habits of families engaged in a healthy eating pilot trial: a thematic analysis

**DOI:** 10.1080/21642850.2022.2043750

**Published:** 2022-02-27

**Authors:** Lucy Porter, Jennifer S. Cox, Kim A. Wright, Natalia S. Lawrence, Fiona B. Gillison

**Affiliations:** a Washington Singer Laboratories, School of Psychology, University of Exeter, Perry Road, Exeter, United Kingdom; b National Institute for Health Research Bristol Biomedical Research Centre, Nutrition Theme, University of Bristol, Bristol, United Kingdom; c Department for Health, University of Bath, Bath, United Kingdom

**Keywords:** COVID-19, child eating behaviour, parent feeding behaviour, COM-B, thematic analysis

## Abstract

**Background:**

The eating habits of children and adults have been impacted by the COVID-19 pandemic, with evidence of increases in snacking and emotional eating, including eating to relieve boredom. We explored the experiences of families with children aged 4-8 years who had recently participated in a healthy eating pilot trial when the first national lockdown began in England.

**Methods:**

Eleven mothers were interviewed in April and May 2020. Interview questions were developed based on the COM-B model of behaviour. Four main themes were constructed using inductive thematic analysis.

**Results:**

The first theme related to an initial panic phase, in which having enough food was the primary concern. The second related to ongoing challenges during the lockdown, with sub-themes including difficulties accessing food, managing children's food requests and balancing home and work responsibilities. The perception that energy-dense foods met families' needs during this time led to increased purchasing of (and thus exposure to) energy-dense foods. In the third theme, families described a turning point, with a desire to eat a healthier diet than they had in the early stages of the lockdown. Finally, in the fourth theme, families reported a number of strategies for adapting and encouraging a balanced diet with their children.

**Conclusions:**

Our results suggest that even if parents have the capability (e.g. knowledge) and motivation to provide a healthy diet for their family, opportunity challenges (e.g. time, access to resources, environmental stressors) mean this is not always practical. Healthy eating interventions should not assume parents lack motivation and should be sensitive to the context within which parents make feeding decisions.

## Introduction

The COVID-19 pandemic has drastically altered the everyday lives of people across the globe, with many countries implementing lockdowns to kerb the spread of the virus. A government-led lockdown commenced in the four nations of the UK at the end of March 2020, with the instruction to stay at home as much as possible, save for one daily excursion for exercise or to purchase food and other essentials (Paun, Sargeant, & Nice, 2020). Schools, offices, restaurants and ‘non-essential’ shops closed, causing substantial disruption to everyday lives and routines. The current study explores the experiences of families with children aged between 4 and 8 years who were already engaged in a healthy eating pilot trial, with a focus on how parents managed to encourage their children to eat a healthy diet during the early stages of the pandemic (if at all).

The disruption of lockdown and other restrictions has impacted the way the public obtain and consume food in a number of ways (Food Standards Agency, 2020b). The increase in time spent at home and the closure of restaurants and other food outlets during the first lockdown meant that more meals needed to be prepared and consumed at home, with research suggesting that the increase in home cooking observed in the first lockdown remained stable in the following months (Food Standards Agency, 2020a). In anticipation of the March 2020 lockdown, both the number of shopping trips and the amount purchased per trip sharply increased in the UK (Kantar, 2020b), which resulted in temporary food shortages in supermarkets across the country. Despite media narratives focusing on the idea of some individuals engaging in antisocial hoarding behaviour, it is likely that this was actually due to small increases in the number of items purchased by the majority of shoppers (Kantar, 2020a), arguably a rational response to an approaching lockdown that would have a substantial impact on eating habits and opportunities for food acquisition (Kantar, 2020b).

A number of studies have also documented the impacts that the COVID-19 pandemic has had on eating behaviour. The majority of findings suggest negative impacts of lockdown measures (although some positive findings have been observed regarding reductions in takeaway consumption; Food Standards Agency, 2020c), with reports of increased intake of snacks and junk food being recorded among adults around the world (e.g. Deschasaux-Tanguy et al., 2021; Di Renzo et al., 2020; Ghosh, Arora, Gupta, Anoop, & Misra, 2020; Husain & Ashkanani, 2020; Robinson, Gillespie, & Jones, 2020; Sidor & Rzymski, 2020; Visser, Schaap, & Wijnhoven, 2020). Weight management behaviours have, in many cases, become more difficult, either due to reductions in the opportunity to exercise or access fresh food, or as a result of the psychological impacts of the pandemic on mental health (Robinson et al., 2021). Some groups have predicted that the conditions required to contain the spread of COVID-19 will lead to a higher prevalence of obesity among both adults and children (Adams, Caccavale, Smith, & Bean, 2020; Robinson et al., 2021). Weight gain in childhood has previously been linked to the school holidays, and researchers have emphasised that the closure of schools during the pandemic may lead to a similar effect as many children spend months, rather than weeks, out of school (Rundle, Park, Herbstman, Kinsey, & Wang, 2020).

Despite these predictions, less evidence has been collected regarding the impact of the pandemic on children’s eating behaviours. The results of the studies that have explored children’s eating behaviours mirror those with adults; a study of children with obesity living in Italy found decreases in self-reported exercise and increases in self-reported intake of energy-dense foods at the beginning of lockdown compared to the previous year (Pietrobelli et al., 2020), while a survey of parents in France revealed increases in snacking and emotional eating among children, with child boredom also being associated with snack intake (Philippe, Chabanet, Issanchou, & Monnery-Patris, 2021). Parents who were highly stressed in this study were also more likely to grant autonomy over eating to their children, which dovetails with evidence for an association between parental stress and children’s intake of energy-dense foods (Parks et al., 2012) and the authors called for qualitative research to explore the reasons for and experiences of these changes (Philippe et al., 2021). A qualitative study with parents of pre-school children in the UK recently investigated the ways in which the pandemic has affected younger children’s (aged two to four years) eating behaviour (among other health-related behaviours such as exercise and screen-time; Clarke et al., 2021). Parents in this study reported a number of negative outcomes, including increased snacking by their children due to numerous factors such as parents giving in to requests more often, children and families being bored whilst staying at home during lockdowns, and using food as a treat in the absence of other opportunities to reward or entertain their children. The present study will expand this to explore the experiences of parents with children aged four to eight who were already enrolled in a healthy eating intervention pilot trial.

It could tentatively be assumed that families’ engagement with a healthy eating pilot trial is indicative of parents’ motivation to improve the eating habits of their children. However, the COM-B model of behaviour specifies that motivation alone is not sufficient in order for a behaviour to occur; the actor must also have the capability (including knowledge and skills) to perform it and the opportunity (including having the time, money, equipment/resources and necessary social environment) to do so (Michie, van Stralen, & West, 2011). Many interventions for improving diet focus on education and building skills (i.e. capability; Johnson, Zarnowiecki, Hendrie, Mauch, & Golley, 2018; Marteau, Hollands, & Fletcher, 2012), and there is a strong rhetoric surrounding obesity that focuses on personal willpower and individual responsibility (i.e. motivation; Brownell et al., 2010; Ulijaszek & McLennan, 2016). The pandemic poses a unique chance to explore what happens when caregivers (who otherwise have the capability, opportunity and motivation to engage in efforts to encourage healthy eating with their children) experience disruptions and stressors brought about by the external environment.

The aim of this study was to explore how these families managed their goals of achieving a healthy diet for their children within the context of the first UK lockdown in Spring 2020. The COM-B model was used to develop the topic guide for the interview in order to ensure that a broad spectrum of influences on behaviour was discussed. Ethical approval for the study was granted by the CLES Psychology Ethics Committee at the University of Exeter (reference number: eCLESPsy001128 v3.3).

## Methods

### Participants and recruitment

Participants were parents or caregivers who were already enrolled in an ongoing pilot trial to test the feasibility and efficacy of a healthy eating app (FoodT^1^) with their children at the time of the March 2020 lockdown. The inclusion criteria for parents to enrol in the pilot trial were (i) parent of a child aged 4–11 years, (ii) resident within 90 minutes travel time (on public transport) of the lead researcher’s address in South London, and (iii) owner of an iOS or Android smartphone/tablet that is compatible with FoodT. Recruitment for the trial was conducted via social media, with adverts being posted on various Facebook groups for parents based in London (predominantly South London). Adverts promoted the pilot trial as an opportunity for parents to test a new app that could increase their children’s intake of fruit and vegetables in exchange for Amazon vouchers. The pilot trial involved two face-to-face visits with a researcher (baseline and two-week follow-up, at which the main study measures were obtained) and an opportunistic follow-up at eight weeks to obtain further feasibility and acceptability feedback from parents after a longer period of using the app. More information about trial procedures can be found at https://osf.io/3h62m. Parents enrolled in the pilot trial were invited to take part in interviews at the eight-week follow-up stage in exchange for a £10 Amazon voucher. Upon the initiation of the March 2020 lockdown, the topic guide for this interview was adapted to include additional questions about healthy eating as a family during lockdown (see below for more details) and those parents whose eight-week follow-up fell after this point (*n* = 22) were eligible for inclusion in the present study.

### Topic guide and data collection

A topic guide was developed for use during data collection (Supplementary File 1). This was added to an existing interview schedule that focused on families’ experiences of the FoodT app and trial procedures (in line with the original aims of the pilot). The topic guide developed for this study included questions that explored, in broad terms, the main changes that parents had experienced or observed regarding both eating behaviour in general (e.g. how has the coronavirus outbreak affected your family’s eating habits?) and achieving a healthy diet as a family specifically (e.g. has eating healthily become harder or easier or is it still the same?). Additional prompt questions were formulated based on the COM-B model of behaviour to ensure that parents were prompted to consider issues of capability (including knowledge and skills), opportunity (including physical environment, time, resources and social influences), and motivation (including changes in prioritisation of healthy eating) during interviews. For example, prompt questions asked parents whether they had the information they needed to achieve a healthy diet as a family during the pandemic (capability), whether they had the equipment or time required to achieve a healthy diet (opportunity) and whether the importance of healthy eating had changed for them (motivation).

LP conducted and recorded interviews with parents via telephone or Zoom (audio only). Participants had consented to the usage of their data for analysis and publication (including consenting to excerpts being published in an eventual journal report) when enrolling in the pilot trial. Parents were reminded of data usage plans and were given the opportunity to withdraw at the start of the interviews if they did not wish for their data to be used in this way. Interviews were conducted between 9th April and 19th May 2020, meaning that all interviews took place during the first lockdown in England. Three parents were interviewed in the week following the announcement that unlimited outdoor exercise and sitting in parks would be permitted (provided these activities were carried out with members of one’s own household only), and garden centres would reopen. Schools and workplaces remained closed during this time (except for key workers and their children). Demographic information, and information about children’s intake of fruit and vegetables and energy-dense snack foods were collected as part of the pilot trial procedures.

### Data analysis

A thematic analysis with an inductive, realist approach was conducted by LP and JSC, with codes and themes being generated at the semantic (explicit) level (Braun & Clarke, 2006). While the inductive nature of this analysis means that no theory of health behaviour was selected as a guiding framework for analysis, both analysts have a background in health psychology and behaviour change, and are therefore likely to have been influenced by pre-existing knowledge of theories in this area.

Before coding, the authors met to discuss the research questions and what ‘types’ of code would be generated to answer the question. Specifically, it was agreed that while the research question primarily focused on how families navigated healthy eating during the pandemic, any responses that pertained to changes in eating habits more widely would also be captured in coding. In addition, there was a particular (but not exclusive) emphasis on generating codes that represented barriers or facilitators to achieving a healthy diet. LP and JSC coded the full dataset independently before meeting to compare the lists of codes that they had generated, noting similarities and differences and working to combine these lists to create a master copy. Where differences were observed (e.g. a code created by one analyst but not the other), these were discussed and resolved (e.g. by adding that code to the master list or by breaking it down and assigning the data extracts to other codes).

Next, the analysts independently generated themes and theme maps with an aim to capture a high-level overview of the narrative they perceived in the data and codes. The analysts then met to discuss these theme maps, again comparing similarities and differences. It was found that both analysts had generated highly similar themes, with one solution including two additional themes to reflect temporal elements of families’ responses to the pandemic. This latter solution was retained for progression and codes were then sorted collaboratively into these themes to check for ‘fit’.

LP wrote an initial report to summarise themes. During this process, the definitions of some themes were revised slightly, as confronting their content and meaning led to the redrawing of theme boundaries and the creation of new themes. Five codes were each added to two themes where appropriate. A summary report was presented to all authors for discussion. No further amendments were made.

## Results

### Participants

Of the 22 eligible parents contacted (all mothers), 11 participated in an interview (it should be noted that the interview had been described to parents as an optional extra activity when they originally enrolled in the study). Parents’ self-described ethnicities included White (*n *= 5), Asian (n = 3), Chinese (n = 1), Black (*n* = 1) and Mixed Black/White (*n* = 1), with ages falling in the range of 26–45 years (Table 1). Seven out of the 11 mothers had Bachelor’s or Master’s degrees and the Index of Multiple Deprivation associated with home postcodes ranged from 2nd most deprived decile to the 7th decile. At the start of their participation in the trial (i.e. before any intervention was received) parents reported that their children ate between 05.57 and eight portions of fruit and vegetables per week and between 0.14 and 3.5 portions of energy-dense snacks per day (Table 1).

**Table 1.** Participant demographic characteristics, including parent reported child intake of fruit and vegetables, and energy-dense snack food portions per week (recorded at pilot trial baseline). Participant ID is not included in order to prevent linking of participant’s identities to specific quotes.

	Parent characteristics				Child characteristics		
	Age band (years)	Ethnicity	Education	IMD home post-code	Age	Fruit/veg portions per day	Energy-dense portions per day
1	31–35	Asian	Bachelor’s degree	7	6	0.57	0.14
2	41–45	White	Master’s degree	3	6	8	1
3	36–40	Black	GCSEs	3	6	3.29	3.5
4	31–35	White	Bachelor’s degree	5	7	3.5	1.86
5	26–30	Asian	A Levels	2	4	5	1.86
6	36–40	White	Bachelor’s degree	6	5	5	1.29
7	26–39	Mixed Black/White	Foundation degree	2	4	2	0.57
8	36–40	White	A Levels	2	8	2	2.57
9	41–45	Chinese	Bachelor’s degree	3	8	5	0.5
10	26–30	White	Bachelor’s degree	7	4	4	2.21
11	31–35	Asian	Master’s degree	5	7	5	0.79

Parents had at least one child aged between 4 and 8 years (i.e. those children who participated in the main trial), with some families having younger children in the home too (this information was not formally collected for all families, but was mentioned by some parents during the interviews, and is therefore reported here for additional context). Ten parents provided information about their working situation at the time of interview, with three working as key workers outside the home, one working as a key worker from home, four working from home in other roles, one enrolled as a full-time student and one on maternity leave. One participant was joined by her partner during the interview, who also contributed to the discussion.

### Themes

**Figure 1.**
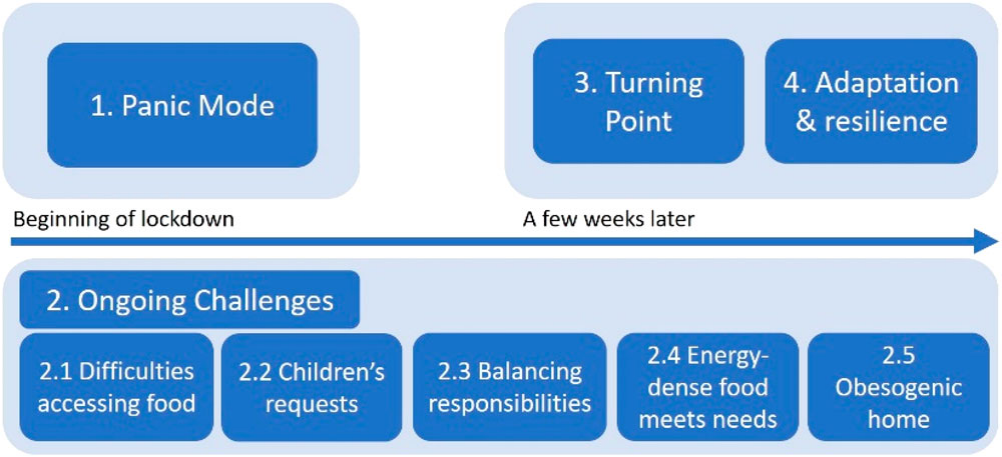
A map of the constructed themes and the time period within which they occurred.

Four main themes were extracted from the data (Figure 1). Two of these had a clear temporal element, with one describing parents’ responses at the beginning of the pandemic (‘Panic Mode’) and the other referring to parents’ reflections a few weeks into lockdown (‘The Turning Point’). The remaining themes did not appear to have a temporal element. One theme (‘Ongoing Challenges’) was broken down into a number of sub-themes to capture the different challenges of life during the pandemic. Some quotes are abbreviated for conciseness or to redact potentially identifying information. Full details of the codes that made up each theme (plus example quotes) are included in Supplementary File 2.

*1.* **Panic Mode: concerns about survival at the beginning of lockdown**. A number of families described how they had gone into panic mode at the beginning of lockdown. During this time, parents described a fear of food shortages and ‘starvation’, which motivated behaviours such as stocking up on foods with a long shelf-life (including energy-dense foods) in order to ensure that there was enough food in the house. One parent referred to ‘surviving’ as being their main priority during the early stages of the pandemic and lockdown. Within this context, discussions around food and shopping/eating behaviours frequently evoked emotions such as panic and anxiety.

… because I was in panic mood which is why I buy a lot of this sugary stuff because if there’s gonna, if there’s gonna be like … […] starvation, we can feed, so we buy rice, we buy some pasta, you know and some sweet stuff in case, you know. (Participant A)

One family mentioned that this included buying ingredients such as pasta and rice; however, many more families described stocking up on energy-dense snack foods such as biscuits and crisps. Some families reported that they typically did not keep certain energy-dense snack foods in the home but that they had changed their usual habits and bought more of these foods during the early stages of the lockdown. For some parents, this was described as being a conscious decision based on the thought that it is better to have energy-dense food than no food at all.

I mean it’s tricky because the only reason we bought junk is because we thought what if there’s no food [laughs] then, then an Oreo cookie is fine. (Participant B)

Relatedly, another reason for healthy habits slipping during the initial lockdown period was the high emotional cost of the situation, meaning that healthy eating was not important in the grand scheme of the pandemic with parents feeling ‘so anxious and worried’. Only one family reported that the initial panic and fear of food shortages at the beginning of lockdown had had a positive impact on the healthiness of their eating habits, due to a conscious decision to reduce portion sizes.

We were rationing because we wasn’t sure what was going to happen [laughs] so our portions became a bit smaller so that was a plus I guess [laughs] yeah. (Participant K)

All parents referred to these changes in the past tense, and overall their responses suggested that these ‘panic mode’ and survival-oriented behaviours had all finished by the time the interviews were conducted.

*2.* **Ongoing Challenges: parents balancing old responsibilities with new demands**. This theme captures the changes to everyday life described by parents, the majority of which caused an increase in demands on parents’ time and energy. Unlike the Panic Mode theme, these issues were described as ongoing at the time of the interview.

2.1. ***Difficulties accessing food*.** The majority of parents described difficulties accessing foods, which was due to both changes in which foods were available (e.g. in supermarkets) and changes to families’ shopping habits in response to the pandemic. Many families reported that they were not able to find the foods they wanted in the usual supermarkets and stores that they used. Online delivery services were also described as being hard to book, and when orders arrived, they were full of substitutions. Consequently, a number of families indicated difficulties accessing required or desired foods, which subsequently impacted whether or not they were able to cook and consume the meals that they wanted to.

Because of my background […] normally we have on a Sunday […] foods like rice and peas and erm … certain, there’s certain ingredients that we need and it’s quite hard to find these ingredients. (Participant D)

Two families mentioned that they had received free school meal parcels. While parents were grateful for the parcels, both of these families indicated that the ingredients provided were not always what they needed. This represents a situation in which issues of food access were not caused by poor availability of food, but by provision of the ‘wrong’ kind of food.

… the hampers we’ve been getting from school, erm they’re kind of a mish mash of things and I think it would’ve been helpful if … well, I mean I say helpful, they’re just trying to put together what they have … (Participant F)

Sometimes, parents described how issues with food availability were compounded by changes in their shopping habits due to pandemic-related infection control practices (such as reducing the number of shopping occasions per month or reducing the number of stores they visited). Three parents’ responses indicated that these altered shopping habits were due to fear of leaving the house altogether rather than compliance with government lockdown guidance (which at the time stated that citizens could leave the house once per day for exercise or essential shopping). Such changes in shopping habits meant that families ran out of certain perishable foods such as fruit and vegetables or could not get hold of the ingredients that they needed to make their usual healthy meals from scratch, which for some families resulted in a reduction in home cooking.

last week I bought so many fruits and it’s finished in a week and now we don’t have anything left and I don’t want to go out just to buy the fruit all the time you know it’s just two times a month I want to go. (Participant A)

In addition to these issues of access and availability, a few parents discussed the increased cost of food shopping as a factor that had changed since the lockdown began. Parents’ responses indicated that this was due to preferred ingredients not being available, meaning that families had to choose more expensive options or go to better-stocked but pricier local stores. While the issues of availability discussed so far predominantly describe negative impacts, a few parents also described how reduced access to some kinds of food had been a positive influence for their family, for example in the reduction of access to takeaways and fast-food due to ‘McDonald’s and things being closed’ (Participant D).

2.2. ***The influence of children’s preferences and requests is magnified during a pandemic.*** Parents’ responses indicated that while the kinds of food children wanted and asked for had not changed much since the pandemic began, navigating and negotiating children’s food requests became more difficult for a number of reasons. Parents reported that their children’s food requests tended to be for energy-dense snacks, and while this wasn’t unusual, the conditions of life during a pandemic meant that these requests were harder for families to deal with (e.g. due to a perceived increase in the frequency of requests or because families were home together all day). Parents’ responses also demonstrated that individual differences between children (in terms of their food preferences or emotional eating styles) were an important factor in the difficulty parents faced when navigating children’s food requests.

One parent reported that requests for energy-dense food had become worse since the beginning of the pandemic due to bad habits developed at the start of lockdown when the focus on just getting by and surviving meant that they were all consuming more energy-dense snacks than usual. This parent also described difficulties getting their children to eat healthier foods such as fruit and vegetables.

there were a lot of tantrums to go through because erm I’ll say in the morning you can have a piece of fruit, yoghurt, you can have … erm we had some cinnamon raisin bagels, those sort of things you can have in the morning when they’re wanting chocolate bars and crisps and things. (Participant F)

In addition, some parents described how their children had reported being hungry more often since the pandemic began, meaning that requests for food had increased overall. This was sometimes described as an additional pressure for parents to contend with, however two parents reported that their child’s increased hunger had led to positive changes, such as greater food intake at meals and greater intake of fruit as a snack. Some parents reported that they had used children’s requests for energy-dense food as an opportunity to negotiate and encourage them to eat something healthy first, and two parents also described their children as actively preferring healthier snacks such as fruit.

yesterday I cut up some fruit and I also give her some party ring and uh she ate all the fruit and said “do I have to eat the party rings?” and I said “no” [both laugh] so you know, she actively chooses not to eat foods um that she doesn’t deem to be healthy. (Participant B)

In addition to hunger, two parents indicated that changes in their children’s mental health had impacted eating habits within the home, with pandemic-related anxiety exacerbating children’s drive for energy-dense foods. One parent described the differences between her two children, with one being ‘in control’ and the other being an ‘anxiety eater’ who was able to ‘eat fifteen packets of biscuits in a row if we let her’ (Participant E). In this case, individual differences in the ability of two siblings to self-regulate had a large impact on the amount of effort their mother needed to expend in order to regulate for them. As well as anxiety due to the pandemic, food availability issues exacerbated problems with children’s food fussiness, with a couple of parents reporting that their children did not like the alternatives that were offered when favoured brands were out of stock.

If it’s any different type of soya he knows the difference and he won’t drink it […] Of all the times to be fussy, you know, I mean … . (Participant F)

2.3. ***Balancing the increased responsibilities of employment and parenthood.*** A number of parents’ responses described increases in workload and responsibility since the beginning of the pandemic, with subsequent impacts on parents’ ability to prioritise healthy eating. While many described saving the time that would have been spent commuting or driving children to after-school activities, these same parents also described an increase in the overall amount they felt was demanded from them on a daily basis. This was due to needing to care for and home-school their children at all times of day while simultaneously managing their usual professional and housekeeping responsibilities. In addition, children’s mental health during the pandemic was mentioned by some parents as a new or heightened concern that they needed to prioritise. One parent described how difficulties coping with these increased demands meant that healthy eating was harder to implement at home, despite having the required knowledge to do it.

I sort of know what is healthy eating so … it’s … it’s not knowing what it is, it’s more like … sort of … during this difficult time, hard to … hard to manage it really with children at home and uh, with the busy household really I do, how do you cope really. (Participant I)

Many parents reported that the additional pressures had led to a worsening of diets within the household (although some parents reported that they were still able to prioritise their child’s healthy eating and that their children’s diets had not changed, even if their own eating habits had become less healthy). Two parents explicitly stated that their own patience for maintaining their children’s healthy eating had occasionally lapsed in the face of trying to balance these additional demands, and that using snacks was a way to help them cope.

I’ve got things I need to do as well so actually sometimes giving them some snacks … is … to keep them quiet, it’s terrible but it’s easier to do when I’ve got to tune into an online lecture or something, it’s not ideal … . (Participant E)

Not all changes in workload and responsibility were experienced negatively. In terms of increased parenthood responsibilities, all parents reported an increase in home cooking, with a number of interviewees stating that this was due to having more time for cooking (e.g. due to no longer needing to commute) – interestingly, some (but not all) of the parents who described the difficulties of balancing work with increased childcare demands also reported having more time to cook and eat together as a family.

we’re not commuting we’re definitely having more time to cook compared to before […] it’s quite nice as a family to all sit down together and eat. (Participant H)

This illustrates one small way in which the changes in working and parenting practices tipped in families’ favour. Overall though, the changes to working and parenting demands described by parents were predominantly negative. Due to these stressors, some interviewees indicated that a healthy eating intervention for families was not what parents needed at this time.

I’ve a lot of friends who are parents and the guilt I think that people are feeling at their children maybe not eating as well, not doing as much homework and all of this stuff is so big at the moment that I reckon if a campaign came out about healthy eating during the coronavirus … I, I don’t know how well it would be received. (Participant E)

2.4. ***Energy-dense food fulfils families’ needs.*** Against these difficulties reported by parents, energy-dense food was often referred to as a quick and easy fix to a number of daily issues. Parents reported using energy-dense food as a treat, eating for comfort, eating to relieve boredom, and for convenience. Firstly, parents reported that food (particularly energy-dense snacks and treats) provided an opportunity to experience something positive and ease negative emotions. Food was described as a way for families to treat themselves in the absence of any other opportunity to do so, and parents reported relaxing their usual restrictions regarding giving themselves and their children energy-dense treats.

We probably are being a bit more relaxed about … um … getting ourselves treats and nice things, […] you feel sorry for the poor little frogs that can’t sort of do much else [laughs] then yeah you just want them to enjoy themselves and be happy so they probably are, they’re probably getting more treats than they normally would yeah. (Participant C)

In addition to being a positive element of families’ daily lives, energy-dense foods were also described as being convenient, and some parents reported using them when balancing the other difficulties of parenting during a pandemic described above. One parent referred to the particular speed of baking cupcakes, another described the ease of buying ready-made nuggets as opposed to making them from scratch in the face of ingredient shortages, and a couple of parents referred to the difficulties of stocking up on perishable foods such as fruit and vegetables versus other foods.

There’s other people who … are trying to … buy on the cheap and to, to buy things cheaply? … to have them last, there are either a lot of frozen items or lots of nuggets and chips, things which you can freeze and, and … and serve bits at a time … erm when you have fresh vegetables they tend to go bad. (Participant D)

Almost half of parents mentioned that they and their children were eating to alleviate boredom, and some parents described cooking and eating as an activity that they could look forward to as a family. While references to using food as an activity or something to look forward to did not exclusively focus on energy-dense foods (e.g. mealtimes were also mentioned), many parents mentioned baking cakes as a fun activity to keep children occupied.

2.5. ***The obesogenic environment at home.*** Following on from the above sub-theme, a number of parents reported an increase in purchasing of energy-dense foods and subsequently, increased availability of these foods in the home. Parents referred to there being more temptations in the house, describing how children had easier access to snacks and that the presence of these foods in the home also increased cravings and requests for these foods (see Section 2.2).

In addition, the disruption of usual routines and increased time spent at home meant that there were fewer options to distract their children from the temptations of the snacks kept at home. Two parents referred to going out for walks as a way to keep their children occupied, also acknowledging that there were fewer opportunities for this kind of distraction due to the need to stay home during lockdown.

If we do go out for a long walk then we, we won’t be taking, we will be taking good snacks with us […] but then if they are at home, they’ve got the temptation a lot more. (Participant I)

In addition, parents reported that spending more time around other family members could sometimes have a negative influence on children’s food environments, either through the purchasing of snack foods or the modelling of unhealthy eating habits.

My husband went to the shop and he brought back four bags just of snacks and I was so [laughs] so cross because I didn’t understand why he wouldn’t buy normal food as well, but he said “you know, the kids eat so many snacks” and I said “well they’ll eat loads of snacks if you keep buying them like that”. (Participant C)

This was not the case for all families. A couple of parents reported that the increased reliance on the home food environment had a positive impact. One parent mentioned that they had healthier foods at home than they would find in food outlets outside the home, and one parent noted that eating all their meals at home had removed the negative influence of their child’s school friends on vegetable intake at mealtimes.

Yeah um I think um [laughs] possibly because he hasn’t got his friends saying ‘eurgh that’s disgusting’ [laughs] um because I think a lot of the time, with school dinners, um, the others around him might not eat specific things, they might um, follow each other. (Participant K)

*3.* **The Turning Point: the increased importance of parents’ healthy eating efforts**. The majority of parents reported what we have described here as a ‘turning point’ some weeks after the beginning of the lockdown. Parents described this moment as being a realisation or heightened awareness of their responsibility for ensuring that their children ate a healthy diet during the pandemic, followed by a desire to either get back to the healthier habits they had before the lockdown or to build entirely new healthy habits to take forward. This tended to be accompanied by parents reflecting on the changes in the family’s eating behaviour over the past weeks, particularly at the start of lockdown.

I thought you know what, we can actually get into a bit of a habit and after two weeks of this awful you know eating whatever, having really random times of meals and I have to say I hadn’t felt hungry in weeks because all I did was eat and then we, we I tried to get a sense of normality back over Easter. (Participant E)

Parents described a number of different motivations underpinning this realisation, one of which was a drive to maintain a healthy weight during a period when activity and exercise were restricted.

It’s about cooking … uh larger meals? [Due to two households merging for lockdown] But making sure that they’re healthier for the, for everyone because of the lockdown and we’re not getting a lot of exercise … We don’t want to be eating a lot of fatty … erm … a lot of fatty foods or a lot of snacks where we’re just piling on the pounds. (Participant D)

Some families also reported that the pandemic had increased the salience of health issues and raised the importance of following healthy habits in general. This prompted them to evaluate their ‘lifestyle and your choices’ and look for opportunities to make changes such as ‘reducing the meat content of our weekly menus’ (Participant B).

As well as these slightly longer-term health goals, the immediate benefits to health and wellbeing (e.g. energy levels and mood) were also described as being important factors for parents after periods of overeating during the first weeks of lockdown. Parents described these changes both in terms of feeling better as a result of eating fruit and vegetables, and avoiding energy rushes in their children (which they couldn’t subsequently ‘burn off’ (Participant H) due to restrictions) as a result of consuming energy-dense foods.

Two parents also highlighted the fact that they were now more responsible than ever for their children’s diets as children being kept home from school meant that parents were the sole provider of meals (as opposed to school dinners, for example). For these parents, the absence of other positive influences (e.g. healthy school meal provision) and the perception that ‘they would have been eating a lot better at school’ was reported as a motivation for building new healthy habits at home (such as ensuring children had at least one portion of vegetables on their plate).

I’m very conscious that they would have been eating a lot better at school and at least then I was like well, I know they’ve had at least one meal or they’ve had some veg because they have to, whereas … at home you’ve got a bit of battle of the wills and erm so I am trying to … you know, at least, you need to choose a veg to go on your plate and stuff whereas before I probably wasn’t as erm … strict about it? (Participant F)

Similarly, a few parents also reported how they had realised the positive potential their own behaviours could have in terms of modelling healthy eating habits for their children, with one parent reporting that they had changed what they ate in front of their child as a result. In these respects, food became something that these families felt that they had control over in an otherwise uncertain and unpredictable world, as illustrated by one parent:

I get to control what we’re eating, it’s important because when you’re in lockdown and we go to the park but you don’t get the same level of exercise, […] I try to be healthy [because] I, I don’t have the benefit of going to the gym anymore so you kind of … it is again back to that thing of what we can control. (Participant G)

Parents who reported these feelings of motivation and control also then described trying new ways to encourage healthy eating in their family, as detailed in the following theme. However, it is important to note that not all parents described the changes in outlook or situation described in this theme.

*4.* **Adaptation and Resilience: parents’ new strategies for achieving a healthy diet**. Parents reported a number of coping strategies to manage the altered everyday reality brought about by the pandemic, ranging from small amendments to cooking and shopping habits (e.g. making use of a farm shop and other local retailers for the first time or trying out a new recipe), to actively using the pandemic as an opportunity to try new approaches to encourage healthy eating with their children. Parents often referred to the need to carry on and get on with things, despite all of the adversities they were experiencing.

it is what it is, things, things have been tough for … hundreds and thousands, thousands of other families, we have each other and you know we still manage to have food and … um … and even if there was something that we would not normally eat um […] you know we might have to go two or three days without um … certain foods but … yeah you just cope don’t you? (Participant G)

However, parents’ responses often indicated additional efforts beyond simply ‘carrying on’, and many described the ways in which they had harnessed new opportunities and tried new strategies to encourage children to eat a healthier diet during the lockdown. Examples of reported strategies include replicating school dinner menus at home, cultivating balcony vegetable patches with children and purposefully ordering mixed vegetable boxes without choosing specific content in order to encourage the family to try something new.

We did like his school menu at home for lunch […] We went through and we said ok Monday, and I did him the same way his school so he had three options and his dessert and then what fruit and veg he was gonna have so then he, we did it together, and he looks at that now as to what he’ll eat and that’s helped a lot actually … . (Participant F)

Some parents also described how they had adapted their food provision behaviours (e.g. by adjusting recipes to accommodate available ingredients, creating new meals using store cupboard ingredients), however, children were not always receptive to these changes.

I think just being adaptable and just kind of saying well sometimes we don’t have what the kids … would like um … if it’s … if we’ve had substitutions of something … and they don’t like it then they don’t, they don’t eat it. (Participant G)

As well as children’s resistance to change, one parent described the difficulties of adapting when they were not always certain which ingredients would be available or what to cook with the ones that were (see 2.1). Another parent also described how new strategies to encourage healthy eating did not always stick and instead ‘petered out’ over a few weeks (Participant F). The majority of parents indicated that they wanted more support and advice about how to encourage their children to eat a healthy diet during the pandemic. Two parents indicated that they had looked to social media for ideas but noted that there was an absence of support for families trying to eat healthily during the pandemic. Parents emphasised that messages would need to be framed in a sympathetic and understanding way, and that any support should be based upon providing families with tools and resources to use rather than simply telling them what to do and adding to their workload.

Most children are not going to substitute … erm you know … crackers and … and erm … cucumber for, for, I don’t know, the cake they’re eating but actually there are ways to make it healthier by reducing the amount of sugar but, you know, […] giving people the options to still do what they want to do but try and make it a little healthier and I think that when things are still phrased in a way that sort of feels that they’re on your side it’s much more, people are much more open to it. (Participant E)

## Discussion

This study set out to explore the ways in which UK families’ eating behaviours, with a specific focus on healthy diets, had been affected by the coronavirus pandemic and national lockdown of March 2020. In particular, the study focused on a group of parents who had already made efforts to improve their children’s diets, as evidenced by their participation in a pilot trial of a healthy eating app from which this study was recruited. For most participants, the early stages of lockdown resulted in a temporary panic and focused on survival amid fears of food shortages and starvation, driving purchases of energy-dense foods. While the more extreme of these fears, fortunately, did not come to fruition for these families, some shortages of specific foods were experienced, which were compounded if parents reduced the frequency of their shopping trips (either in accordance with government guidelines or due to family’s own fear of infection). Families also found themselves facing additional new challenges, including balancing the demands of home schooling, managing children’s mental health, cooking more meals than usual and working from home.

Against this backdrop, parents’ responses painted a picture of a group of people trying to do the best they could for their families in a period of immense change and stress. Parents wanted their children to eat a healthy diet, but this wasn’t always something that they could prioritise due to the demands of everyday life becoming more difficult and time-consuming for them. Sometimes, parents felt that doing the best for their children simply meant ensuring that there was food in the cupboards, even if this food was a biscuit. Other times, energy-dense foods were chosen because they were a small way that children (and parents) could enjoy a treat in the otherwise dull world of lockdown. Sometimes, parents used these foods because their convenience enabled them to balance meal provision with other demands such as home schooling or reducing trips to the supermarket in order to protect themselves from infection. A couple of parents openly acknowledged that the challenging circumstances had led to them being more permissive or giving in to children’s food requests, an understandable (and potentially very common) strategy for coping in the difficult circumstances. Many of these changes (including eating for boredom or using energy-dense foods as a treat) were also reported in a recent qualitative study on the experiences of parents with pre-school children (Clarke et al., 2021), while increases in parental purchasing and provision of energy-dense foods were also found in the studies of Adams et al., 2020; Philippe et al., 2021 and Snuggs & McGregor, 2021. The current findings provide greater insight into the motivations and experiences underpinning parents’ feeding decisions.

Despite the challenges associated with the pandemic and the lockdown in particular, parents also recognised the importance of their role as providers of healthy food and engaged in a number of different strategies to keep healthy meals on the table and to instil healthy habits in their children. In many cases, this appeared to be triggered by a moment of reflection a few weeks into lockdown, with the majority of parents describing the point at which they had realised that their family’s eating behaviour needed to change (e.g. due to lower exercise, higher salience of health or poor eating habits during the first weeks of lockdown). This increase in the importance of health during or after lockdown mirrors findings from other studies with adults (Marty, de Lauzon-Guillain, Labesse, & Nicklaus, 2021; Snuggs & McGregor, 2021). Despite the limited opportunities for distraction and entertainment, parents reported resourcefulness and creativity in their strategies for encouraging healthy eating with their children. One parent described recreating school meals at home to ensure children were eating a balanced diet with fruit and vegetables, and another spoke about the vegetable patch that she and her child were cultivating on the balcony of their flat.

For the families in this study, the early stages of the pandemic and lockdown represented an acute period of distress with a rise in competing priorities when making food decisions, and these findings, therefore, emphasise the importance of context on individuals’ ability to achieve a healthy diet for the family. Other research with individuals experiencing food insecurity also demonstrates that difficulties with accessing food (e.g. due to cost) and the effort required to manage competing priorities also denies people the luxury of focusing on health when choosing food (Puddephatt et al., 2020). A relationship between parental stress and child intake of fast-food has also been observed previously (Parks et al., 2012), as has a relationship between parental stress and indulgent feeding behaviours (Loth, Uy, Neumark-Sztainer, Fisher, & Berge, 2018), which dovetails with the finding here that parents were more likely to give in to children’s food requests and purchase convenient energy-dense foods when stressed or short on time as a way of getting by. The finding that fears of food shortages and hunger led some families to break existing habits of not keeping energy-dense foods in the house also aligns with previous research with low-income parents, which found that fears of child hunger drove parents to provide children with foods that they felt guilty about providing (Pescud & Pettigrew, 2014). While parents in the current study reported using strategies to get their families back on track with healthy eating goals, there is a risk that for families who do not have the capacity to reprioritise healthy eating (or prioritise it in the first place), a deterioration in health behaviours could turn into longer-term habits. Others have described the combination of food insecurity and the pandemic as a ‘double whammy’ for childhood obesity (Tester, Rosas, & Leung, 2020), which is concerning given that food insecurity has increased since the pandemic began, with households with children being more likely to suffer from food insecurity than those without (Food Standards Agency, 2020c).

### Application of the COM-B model in interpreting findings

The COM-B model of behaviour specifies that in order for a behaviour to occur, the actor must have the capability, opportunity and motivation to perform it (Michie et al., 2011). The largest changes brought about by lockdown were observed in terms of parents’ opportunity to provide a healthy diet for the family. A reduction in opportunity to provide healthy food was related to; changes to the demands placed on parents and the amount of time available to them (i.e. working from home, schools being closed requiring more time spent on child care), difficulties accessing food as a result of temporary shortages, reduced frequency of shopping or accessing different shops than usual and in many cases, greater opportunity for the consumption of energy-dense foods due to temporary changes in shopping patterns and relaxation of usual food ‘rules’ changing the food environment within the home. On the other hand, some parents found there was greater opportunity for healthy eating through having more time to cook, or engaging children in cooking and trying new things.

With regards to capability (e.g. knowledge and skills), parents indicated that whilst they knew how to provide a healthy diet for their children normally, some found this difficult to apply in the context of the changes brought about by the pandemic. While only one parent explicitly stated that they didn’t know how to cook with the ingredients available to them, many more reported that there was a general lack of support or information on how to keep children healthy at home during the pandemic lockdown measures Parents indicated that any such informational interventions or resources should be provided in a supportive and sympathetic manner that openly acknowledged the difficulties parents were experiencing, and that instructions on what they ‘should’ be doing would not be received well.

Within our participant group, there was evidence that parents’ motivation towards ensuring their child ate a healthy diet may have changed during the pandemic. While many parents reported that healthy eating was still important to them, these motivations were often challenged by conflicting goals. For example, in most cases, parents’ initial responses to the lockdown was to buy foods with long shelf-lives (which sometimes meant buying energy-dense foods such as biscuits) in order to protect against food shortages. After the initial phase, parents were sometimes still motivated to buy more energy-dense foods to meet particular needs (e.g. treats, convenience) or to allow greater permissiveness with energy-dense food requests as a treat to children upset by the changes to their lives, and to balance the demands placed on them throughout the day. There were also discussions of automatic drivers such as fears of starvation and managing emotions such as stress and anxiety. However, for the majority of participants, motivation shifted to focus more on long-term health once parents felt the initial crisis was over.

Assessing behaviour using the COM-B framework can help to identify whether all of the necessary conditions for behaviour are in place (Public Health England, 2020). In this case, it is revealing that the circumstances of the pandemic resulted in a number of unhealthy behaviours among interviewed families, caused by opportunity challenges, capability difficulties and competing motivations. The parents interviewed here were likely to be highly-motivated (inferred by their participation in a healthy eating trial), and given that these families were sufficiently challenged by the difficult circumstances of the pandemic that they could no longer focus on providing a healthy diet, even if only temporarily, this suggests that intervention approaches based on information-provision, persuasion or narratives of personal responsibility are unlikely to be effective, acceptable or fair. This was echoed in parents’ responses, with one mother suggesting that any messages aimed at parents should be framed in an understanding way that showed that message sources acknowledged the challenges parents were facing.

### Limitations

This study was conducted with a small sample of 11 parents, meaning that their experiences are unlikely to exhaustively represent those of parents in the UK during this time. In addition, the parents who were interviewed for this study were likely to be motivated to improve their children’s diets (as inferred by their participation in the original pilot trial), so although the barriers they experienced to eating a healthy diet may have been common to others, their efforts to overcome these may not be representative of the experiences of the wider population. In addition, while none of the families in this study reported income reductions or job losses, other data indicates that this is an adverse circumstance affecting a high number of households within the UK (Connors et al., 2020; Food Standards Agency, 2020c). Others have described how coping strategies described here, such as stocking up on foods with a long shelf-life, may not be affordable for those living with food insecurity (Tester et al., 2020). Therefore, the findings of this study may not reflect the experiences of many other families. Even within this sample, not all parents reported a ‘turning point’ or trying new strategies to encourage healthy eating with their children meaning that some families had not reached a point where they felt able to reprioritise healthy eating in their family due to the other demands placed on them.

An additional limitation is that interviews were conducted during the first UK lockdown during the Spring of 2020. Since then, the nations of the UK have gone through further lockdowns, including a graduated system that saw different areas of England placed under ‘tiers’ of measures. It is possible that families’ situations and behaviours will have evolved over this longer time period, and further work is needed to understand how eating habits have adjusted over time. Periods of change (such as moving house) have previously been found to be a prime opportunity for the disruption of old habits and the development of new ones (Verplanken & Wood, 2006); additional research is needed to explore which behaviours were maintained by families and which were not against the backdrop of continuously changing measures to contain COVID-19.

Finally, this study was an opportunistic one, with the current topic guide being developed rapidly to fit with an existing interview schedule on short timeframes. As demographic data had already been collected for the main pilot trial, we did not formally collect additional data on family characteristics that may have been useful for contextualising these findings (e.g. working status, number of children in the household). We contacted parents by email to see if they would be willing to provide this information, but unfortunately did not hear back from all families, meaning that contextual data is incomplete.

## Conclusions

Parents in this study reported a number of significant changes to their family’s eating habits, including numerous severe barriers to providing a healthy diet. While the majority of parents in this study also reported positive changes and the implementation of new strategies to improve their children’s diet, not all families reached this point. At the time of writing, the coronavirus pandemic is still ongoing, with a recent lockdown in the UK resulting in primary education being moved to the online setting for the second time. Our findings suggest that families who are still ‘fighting fires’ such as increased workload and heightened stress, all whilst managing the needs of their children, may not find it practical to engage with healthy eating efforts during these periods. Focusing on personal responsibility and willpower will not be sufficient or fair; parents in these circumstances need compassionate support to overcome the barriers to healthy eating caused or exacerbated by the pandemic.

## Note

1
http://www.exeter.ac.uk/foodt
 
